# The effects of cumulative stressful educational events on the mental health of doctoral students during the Covid-19 pandemic

**DOI:** 10.14324/111.444/ucloe.000048

**Published:** 2022-11-08

**Authors:** Vassilis Sideropoulos, Emily Midouhas, Theodora Kokosi, Jana Brinkert, Keri Ka-Yee Wong, Maria A. Kambouri

**Affiliations:** 1 IOE (Institute of Education), UCL’s Faculty of Education and Society (University College London), Department of Psychology & Human Development, UK; 2 Department of Population, Policy, and Practice, UCL Great Ormond Street Institute of Child Health, University College London, UK

**Keywords:** Covid-19, doctoral students, educational experiences, mental health, stressful events

## Abstract

High rates of psychological distress including anxiety and depression are common in the doctoral community and the learning environment has a role to play. With the coronavirus disease (Covid-19) pandemic taking a toll on mental health it is necessary to explore the risk and protective factors for this population. Using data from the Covid-19: Global Study of Social Trust and Mental Health, the present study examined the relationship between Covid-19-related stressful educational experiences and doctoral students’ mental health problems. Moreover, it assessed the role of attentional ability and coping skills in promoting good mental health. One hundred and fifty-five doctoral students completed an online survey where micro-, meso- and macro-level educational stressors were measured. The Patient Health Questionnaire and the Generalized Anxiety Disorder Questionnaire were used to measure depression and anxiety symptoms, respectively. We also measured coping skills using a 13-item scale and attentional ability using a questionnaire. The results of multiple linear regression analyses showed that specific stressful educational experiences were unrelated but cumulative stressful educational experiences were related to increased depression symptoms (but not anxiety symptoms) in fully adjusted models. Additionally, higher coping skills and attentional ability were related to fewer depression and anxiety symptoms. Finally, no associations between demographics and other covariates and mental health problems were found. The experience of multiple educational stressful events in their learning environment due to Covid-19 is a key risk factor for increased mental illness in the doctoral community. This could be explained by the uncertainty that the Covid-19 pandemic has caused to the students.

## Introduction

The lead question for this series of studies was: How has the coronavirus disease (Covid-19)-altered environment impacted health and relationships? In learning environments, such as universities, access to resources became limited and research student projects were often grounded to a halt, if not moved to a virtual environment. This study aimed to examine the wellbeing of doctoral students. A growing body of psychological and psychiatric evidence reveals that the impact of the Covid-19 pandemic on mental health has become of increasing global concern [1]. Similarly, the World Health Organization has expressed concerns over the impact of the pandemic on the psycho-social aspects of life [2]. A recent systematic review and meta-analysis comparing data prior to and during the Covid-19 pandemic [3] documented a moderately small increase in mental health problems during the outbreak of the pandemic, however, mental health problems remained either high or stable by mid-2020 for most populations [3,4]. Nonetheless, slight differences have meaningful cumulative consequences at the population level and for specific groups. For instance, there is evidence suggesting that those with pre-existing mental health problems (mood disorders such as depression) were at a higher risk of problem increases during the Covid-19 pandemic [4,5]. Such a high-risk subgroup is that of doctoral students. In recent years, doctoral students’ mental health has become a focal topic in educational research due to alarmingly high rates of clinical symptoms experienced by doctoral students [6] and the consequences of mental health disorders on doctoral students’ completion rates [5,7]. Previous studies have reported that one in three doctoral students is at risk for a common psychiatric disorder [5], with anxiety and depression being six times higher amongst doctoral students compared to the general population [7]. There have been increases in the numbers of individuals pursuing doctorates in recent decades [8] and they are integral to the development of academic research in a broad sense [9,10]. Hence, it is vital, against the backdrop of the Covid-19 pandemic, to understand more about both the vulnerability of doctoral students and what factors might promote good mental health amongst them.

Stress related to educational challenges experienced by doctoral students may be the reason for their vulnerability to mental health problems. Given the time required, the need to produce scientifically rigorous and independent research that meets a high standard and the importance of good supervision for success, the doctoral process can be gruelling and lonely for some. Specific educational factors that have been associated with worsening doctoral students’ mental health include: a) supervisory problems which can lead to personal or professional conflicts [11]; b) limited access to resources such as the lack of support from the department they are hosted in [12]; c) domain-specific expertise, including the lack of supervisor and student knowledge in mental health which can result in students being insufficiently supported [13]; d) lack of competence with general work processes which most doctoral students face as they embark on a PhD/Professional Doctorate degree straight after their academic training [13]; e) external or personal challenges such as moving houses or experiencing family problems [7,14]; and f) project-related challenges such as intellectual property disputes [14].

Of those experiencing mental health distress, one in three are hesitant to seek access to institutional advice and support services in the UK; some reasons are the lack of signposting to mental health services in universities as well as the lack of parity from higher education support services [5,6,13,15]. The lack of access to non-academic support (e.g., personal and/or pastoral support) for mental health could lead to an accumulation of personal and professional adversities [16], a key question for investigation in this study. Previous research has only looked at specific single risk factors associated with doctoral students’ mental health [5,6,13,15]. A large body of research on stressful life events has indicated that the accumulation of risk is more important than specific single factors risk for mental health problems [17]. While researchers have investigated different institutional- and individual-level factors that could provide insight into doctoral students’ mental health, research on the link between cumulative and global factors and mental health is limited. This is particularly important as evidence from the mental health literature suggests that, rather than specific types of individual events, the accumulation of multiple adverse experiences have a worse effect on people’s mental health [18,19]. Numerous studies have documented the cumulative effects of multiple stressful events experienced by a person in the general population and their association with mental health [18]. For instance, there has been work showing the impact of cumulative exposure to poor housing can have adverse effects on mental health and wellbeing [20]. Similarly, psychiatric and clinical studies used this approach to understand the impact of cumulative childhood trauma on mental health [21] as well as the accumulation of physical, psychosocial and health adversities’ impact on academic achievement of children [22]. In addition, findings from a psychiatric report indicated that the cumulative effects of life events have an impact on both physical and mental health [19] and certain circumstances of life, such as workload and changing patterns of familiar meetings, can cause mental health turbulence. Thus, the accumulation of multiple adverse experiences during the pandemic may be predicted as an added risk for subgroups of individuals. Yet little is known about the role of cumulative stressful educational factors in the mental health problems of doctoral students. The extent to which doctoral students experienced these factors as well as how such factors, taken together, jointly affect mental health problems, is unclear.

Moreover, there is limited evidence of factors that might promote mental health in the doctoral student population. There are two factors – coping and attentional skills – that show promise in terms of being able to promote good mental health in doctoral students. There is evidence that training in coping skills – cognitive or behavioural strategies used to reduce negative emotions due to stressors – can be effective when it comes to the maintenance of wellbeing and good mental health [20] particularly for those with anxiety-related disorders. Yet not much is known about the role of coping in depression and social dysfunction disorders for doctoral students. Whilst there is some research examining the relationship between coping skills and depression in undergraduate and graduate student populations (which primarily includes master’s students, [21–23], to the best of our knowledge, no research has explored coping skills amongst doctoral students. Likewise, the role of attention and its relationship with anxiety and depression has not been addressed either in the doctoral literature despite the evidence showing that better attentional control skills are likely to promote better mental health in college students [24]. Identifying both risk and promotive factors may help university services to offer better support to students in the future. Taken together, investigation into doctoral students’ mental health should be based on multidimensional frameworks that account for diverse and multiple factors that may affect one’s emotional state.

Epidemiologists and mental health researchers have used different methods and techniques to study mental health along with the prevalence and risk factors by using advanced and complex statistical approaches that can account for several factors such as socio-economic background, education, gender differences and more [25]. In this current work, we focus on the accumulation of adversities and their impact on mental health in doctoral students within the context of the Covid-19 pandemic and its associated educational challenges. In addition, we take into consideration the challenges of doctoral students through an ecologically inspired framework where the challenges that lead to poor mental health derive from several educational contextual as well as individual factors. These could be related to institutions’ structures and policies, the relationships between students and academic staff as well as students’ interpersonal relationships and individual characteristics [26]. This is particularly meaningful given that previous research has shown how synergistic approaches to mental health allow for better understanding and help prevention and relapse [27]. In addition, understanding the challenges that doctoral students face offers ways to mitigate difficulties [12]. However, an integrated approach to doctoral students’ mental health is yet to be operationalised in research.

Consequently, the purpose of this study is to explore the effect of specific and cumulative stressful educational events (CSEE) on doctoral students’ mental health during the Covid-19 pandemic. Specifically, it examined whether doctoral students’ mental health problems (anxiety and depression) are affected by both specific and multiple stressful events (rather than specific types of single events) ranging from interpersonal characteristics to institutional policies as well as exogenous factors such as the impact of Covid-19 on the students. In this paper, we use the sum of stressful educational events in an analogous way to mental health research in other fields [21–23].

### The present study

The aim of this study was to explore the impact of an accumulation of multiple stressful events (CSEE), on doctoral students’ mental health during the Covid-19 pandemic. We also adjust for a range of variables that may confound the relationship including education contextual factors (whether PhD students belong to a research lab) and individual-level factors (funded vs. self-funded, age and ethnicity). Furthermore, we explore the relationship of coping and attentional skills as factors that may promote good mental health.

## Methods

We used data from the longitudinal Covid-19: Global Study of Social Trust and Mental Health [28], from Wave 2 when survey data were collected between 17 October 2020 and 31 January 2021. The data were collected using an anonymous survey that was distributed via Qualtrics, an online survey tool. Further details on study methodology can be found elsewhere (https://osf.io/fe8q7/).

### Participants

For this paper, we only considered participants who provided complete responses on the mental health scales. One hundred and fifty-five doctoral students (80.9% female) aged 23 to 69 years [mean = 30.24, standard deviation (SD) = 7 years] completed the online survey. The majority of participants were in their 2^nd^ year of studies (n = 39) at the time the survey was completed. A more detailed breakdown of the demographic and educational variables of our sample is presented in Table 1. A full list of countries where responses have been drawn from are available in Supplementary Materials, Supplementary Table 1.

**Table 1.** Demographic and educational variables by n of cases and percentages

Demographic and covariate variables	n	%
Age (years)				
18–24	11	8.1
25–34	103	75.7
35–44	14	10.3
45–54	5	3.7
55+	3	2.2
Gender				
Female	123	80.9
Male	29	19.1
Ethnicity				
White	103	68.2
Non-White	52	31.8
Year of studies				
First year	38	26.2
Second year	39	26.9
Third year	31	21.4
Fourth year	21	14.5
Fifth year	12	8.3
Sixth year	4	2.8
Part of a research group				
Yes	102	70.8
No	42	29.2
Funded				
Yes	34	23.4
No, self-funded	111	76.6

All percentages presented in Table 1 are Valid Percent (Missing data are excluded from the calculations)

The participants were recruited through social networks and word of mouth. Anyone above the age of 18 with access to the study link was eligible for the main Covid-19 study. In our study, we considered only those participants who stated that they were currently studying for either a Doctor of Philosophy (PhD) or a Professional Doctorate degree. Participants who reported that they were a doctoral student were shown an extra set of questions about their doctoral experience and the challenges they faced thus far through open-ended and closed questions.

### Materials

A list of the measures used in the survey can be accessed freely on the OSF website [28] (https://osf.io/fe8q7/). In the current study, we examined data from four questionnaires, demographic questions and other open-ended and closed questions which can be found below.

#### Mental health

The 9-item Patient Health Questionnaire (PHQ-9) [29] which uses a 4-point scale (not at all [0], several days [1], more than half the days [2], nearly every day [3]) was used to assess depressive symptoms. A high score denotes higher levels of depressive symptoms with a score of 15 being the clinical cut-off. We calculated the reliability of our scales, Cronbach’s α = 0.88 for both unstandardised and standardised measures.

The 7-item Generalized Anxiety Questionnaire (GAD-7) [30] which uses a 4-point scale (not at all [0], several days [1], more than half the days [2], nearly every day [3]) was used and high summed scores reflect higher levels of anxiety. The clinical cut-off point for GAD-7 is a score above 15. Reliability was also calculated for this scale; Cronbach’s α = 0.91 for unstandardised and α = 0.90 for standardised.

#### Coping skills and attentional abilities

The 14-item Coping Skills Questionnaire [31] which uses a 4-point scale (not true about me [1], a little true about me [2], somewhat true about me [3], mostly true about me [4]) and was used to assess cognitive, emotional, and behavioural methods of dealing with problems. Higher summed scores indicate higher levels of coping. Cronbach’s α = 0.81 for both unstandardised and standardised.

An adapted 7-item version of the 18-item Adult Attention-deficit/hyperactivity disorder (ADHD) Self-Report Scale (ASRS-v1.1) [32] which uses a 5-point scale (never [0], rarely [1], sometimes [3], often [4], very often [5]) to assess lower attentional focus. Higher summed scores indicate lower levels of attentional focus. For this scale, Cronbach’s α = 0.78 for unstandardised and α = 0.79 for standardised.

#### Cumulative stressful educational events

CSEE were measured with a newly developed composite variable based on the total number of events experienced. Events were: research impacted by Covid-19, interruption from PhD, forced adaptation to research, supervisor change and other problem. To create the cumulative variable, we used the total score of those binary variables, and the maximum number of stressful educational events was 5. Table 2 presents the exact questions along with the n of participants per answer as well as the percentages.

**Table 2.** Characteristics of the stressful educational events collected from the sample prior to summing up as a cumulative variable

Cumulative stressful educational events	n	%
Is there any impact on your research because of Covid-19?				
Yes	84	67.7
No	40	32.3
Did you interrupt your PhD?				
Yes	13	10.4
No	112	89.6
Did you have to make any adaptation to your research projects?				
Yes	65	52.0
No	60	48.0
Did you have to change a supervisor in the last 6 months?				
Yes	12	9.6
No	113	90.4
Is there any other problem you’ve experienced?				
Yes	23	20.0
No	92	80.0

#### Covariates

Participants reported their age, gender, ethnicity, whether they are part of a research group and whether they are funded/self-funded students. These variables, apart from age, were then categorised into binary variables and were included in our analyses as covariates; ethnicity (White vs. Non-White); gender (female vs. male); part of a research group (yes vs. no); funded (yes, funded vs. no, self-funded).

### Ethics

Ethical approval for the study was obtained from the Ethics Committee of UCL Institute of Education prior to the data collection (REC 1331, REC 1345). Respondents provided online consent to participate in the study and to be followed-up.

### Data analysis

First, we described mental health of our sample using descriptive statistics. Next, we ran a series of linear regression models for each mental health outcome – anxiety and depression. The first model had the cumulative events as the main independent variable. The second model adjusted for the socioeconomic and educational covariates. The third model added the two individual-level variables that we expected would promote mental health, coping skills and attentional ability. Therefore, we ran a total of six models. We present the models for the specific events in Supplementary Tables 2 and 3 as there were no significant associations of the specific events with the mental health outcomes and the scope of the paper was the accumulation of stressful educational events.

## Results

### Descriptive statistics

The data show that a small proportion of the doctoral students (14.28%, n = 18) scored above the cut-off threshold for clinical depressive symptoms and similarly, only a few doctoral students scored above the cut-off threshold for clinical anxiety symptoms (21.43%, n = 19). Table 3 presents an overview of the mental health questionnaires.

**Table 3.** Overview of the mental health questionnaires split into the threshold categories for clinical symptoms

Mental health questionnaires	n	%
Depression				
None–minimal	55	35.5
Mild	56	36.1
Moderate	23	14.8
Moderately severe	14	9
Severe	7	4.5
Anxiety				
Moderate	32	20.6
Mild	88	56.8
Severe	35	22.6

### Predictors of depression

In the multiple linear regression models (Table 4 for coefficients, Table 5 for model output), the experience of CSEE (β = 1.16, *P* < 0.001) is associated with higher levels of depressive symptoms. When adjusted for covariates, CSEE (β = 1.11, *P* < 0.001) and ethnicity (β = 2.44, *P* = 0.05) were associated with higher depressive symptoms. Finally, when adjusted for the cognitive factors, both coping skills (β = ‒0.21, *P* < 0.001) and lower attentional abilities (β = 0.65, *P* < .001) were associated with higher depressive symptoms in the doctoral community by severity of symptoms.

**Table 4.** Coefficients for depression models

								95% confidence interval (CI)	
Model		Unstandardised	Standard error	Standardised	t	*p*	VS-MPR*	Lower	Upper
Model 1 – Depression – CSEE	(Intercept)	5.44	0.96		5.64	1.26e‒7	184282.98	3.53	7.35
	**Cumulative Events**	**1.16**	**0.39**	**0.27**	**2.96**	3.76e‒3**	**17.53**	**0.38**	**1.94**
Model 2 – Depression – CSEE and covariates	(Intercept)	5.48	5.33		1.03	0.31	1.02	‒5.09	16.04
	**Cumulative Events**	**1.11**	**0.41**	**0.26**	**2.73**	7.36e‒3**	**10.18**	**0.31**	**1.92**
	Age	‒0.06	0.10	‒0.06	‒0.58	0.57	1.00	‒0.26	0.14
	**Ethnicity**	**2.44**	**1.24**	**0.19**	**1.97**	0.05**	**2.42**	**‒0.01**	**4.89**
	Gender	‒5.20e‒3	1.50	‒3.22e‒4	‒3.46e‒3	1.00	1.00	‒2.98	2.97
	Part of a Group	‒1.90	1.29	‒0.15	‒1.47	0.14	1.32	‒4.45	0.66
	Funded/self-funded	0.63	1.40	0.04	0.45	0.66	1.00	‒2.16	3.41
Model 3 – Depression, CSEE, covariates and cognitive factors	(Intercept)	‒1.74	5.63		‒0.31	0.76	1.00	‒12.91	9.43
	**Cumulative Events**	**0.74**	**0.34**	**0.17**	**2.18**	0.03**	**3.40**	**0.07**	**1.40**
	Age	‒0.07	0.08	‒0.07	‒0.86	0.39	1.00	‒0.24	0.09
	Ethnicity	0.94	1.05	0.07	0.89	0.38	1.00	‒1.15	3.02
	Gender	0.71	1.24	0.04	0.57	0.57	1.00	‒1.74	3.16
	Part of a Group	0.60	1.13	0.05	0.53	0.60	1.00	‒1.64	2.83
	Funded/Self-funded	0.32	1.16	0.02	0.28	0.78	1.00	‒1.97	2.61
	**Lower attentional abilities**	**0.65**	**0.10**	**0.52**	**6.32**	6.49e‒9**	**3.01e+6**	**0.45**	**0.86**
	**Coping skills**	**‒0.21**	**0.07**	**‒0.23**	**‒3.08**	2.67e‒3**	**23.28**	**‒0.34**	**‒0.07**

*Vovk–Sellke maximum *P*-ratio: Based on the *P*-value, the maximum possible odds in favour of H_1_ over H_0_ equals 1/(-e *P* log(*P*)) for *P* ≤ 0.37.

**Statistically significant coefficients.

**Table 5.** Multiple linear regression outputs

Models	Multiple linear regression outputs
Model 1 – Depression	F(1,114) = 8.76, *P* < 3.76e‒3, R2 = 0.07, R2 adjusted = 0.06
Model 2 – Depression and covariates	F(6,114) = 2.58, *P* < 0.01, R2 = 0.13, R2 adjusted = 0.08
Model 3 – Depression, covariates and cognitive Factors	F(8,113) = 9.67, *P* < 0.001, R2 = 0.42, R2 adjusted = 0.38
Model 4 – Anxiety	F(1,114) = 2.29, *P* = 0.13, R2 = 0.02, R2 adjusted = 0.01
Model 5 – Anxiety and covariates	F(6,114) = 0.73, *P* = 0.63, R2 = 0.04, R2 adjusted = ‒0.01
Model 6 – Anxiety, covariates and cognitive factors	F(8113) = 8.92, *P* < 0.001, R2 = 0.40, R2 adjusted = 0.36

### Predictors of anxiety

For the multiple linear regression models of anxiety (Table 6 for coefficients, Table 5 for model output), the experience of CSEE (β = 0.72, *P* < 0.02) is associated with higher anxiety symptoms only in the null model. When adjusted for covariates, none of the factors were associated with anxiety. Finally, in our last model where we adjusted for the cognitive factors, we found again that low coping skills (β = ‒0.17, *P* < 2.09e‒3) and lower attentional abilities (β = 0.55, *P* < 1.27e+7) were associated with higher anxious symptoms.

**Table 6.** Coefficients for anxiety models

								95% CI	
Model		Unstandardised	Standard Error	Standardised	t	*P*	VS-MPR*	Lower	Upper
Model 4 – Anxiety – CSEE	(Intercept)	4.45	0.77		5.75	7.73e‒8	290554.31	2.92	5.99
	**Cumulative Events**	**0.72**	**0.32**	**0.21**	**2.29**	0.02**	**4.09**	**0.10**	**1.35**
Model 5 – Anxiety – CSEE and covariates	(Intercept)	5.01	4.36		1.15	0.25	1.06	‒3.63	13.64
	Cumulative Events	0.62	0.33	0.18	1.88	0.06	2.11	‒0.03	1.28
	Age	‒0.07	0.08	‒0.08	‒0.80	0.43	1.00	‒0.23	0.10
	Ethnicity	0.41	1.01	0.04	0.40	0.69	1.00	‒1.60	2.41
	Gender	1.44	1.23	0.11	1.17	0.25	1.07	‒1.00	3.87
	Part of a Group	‒0.79	1.06	‒0.08	‒0.75	0.46	1.00	‒2.88	1.30
	Funded/Self-funded	‒0.28	1.15	‒0.02	‒0.25	0.81	1.00	‒2.56	1.99
Model 6 – Anxiety – CSEE, covariates and cognitive factors	(Intercept)	‒1.28	4.55		‒0.28	0.78	1.00	‒10.30	7.73
	Cumulative Events	0.31	0.27	0.09	1.14	0.26	1.05	‒0.23	0.85
	Age	‒0.08	0.07	‒0.09	‒1.15	0.25	1.06	‒0.21	0.06
	Ethnicity	‒0.86	0.85	‒0.08	‒1.01	0.31	1.01	‒2.54	0.83
	Gender	2.02	1.00	0.16	2.03	0.05	2.63	0.04	4.00
	Part of a Group	1.34	0.91	0.13	1.47	0.14	1.32	‒0.47	3.15
	Funded/Self-funded	‒0.54	0.93	‒0.05	‒0.58	0.56	1.00	‒2.39	1.31
	**Lower Attentional Abilities**	**0.55**	**0.08**	**0.56**	**6.64**	1.42e‒9**	**1.27e+7**	**0.39**	**0.72**
	**Coping Skills**	**‒0.17**	**0.05**	**‒0.24**	**‒3.16**	2.09e‒3**	**28.56**	**‒0.28**	**‒0.06**

*Vovk–Sellke maximum *P*-ratio: Based on the *P*-value, the maximum possible odds in favour of H_1_ over H_0_ equals 1/(-e *P* log(*P*)) for *P* ≤ 0.37.

**Statistically significant coefficients.

## Discussion

In this paper, we explored the impact of CSEE on doctoral students’ mental health during the Covid-19 pandemic between 17 October 2020 and 31 January 2021 by operationalising into our model a range of variables from macro–meso–micro level factors related to the university experience. The consideration of multiple variables into our linear modelling is rooted in the evidence that strongly suggests that doctoral students’ mental health should be investigated in a more complex and systematic way [33]. Our statistical approach allows for a better understanding of the specific effects of CSEE on doctoral students’ mental health, specifically anxiety and depression.

Whilst the current pandemic has affected the mental health of much of the population [1–4], our findings show that 28.3% of doctoral students reported mild-to-severe depressive symptoms and 79.4% of them reported moderate-to-severe symptoms for anxiety in our sample. Our findings are in line with previous research conducted prior to the pandemic [6,7,11,15,16] which shows that doctoral students experience high levels of depression and anxiety. Furthermore, our findings align with other research that suggests an increase in mental health difficulties in doctoral students’ during the Covid-19 pandemic [34].

As part of our second statistical analyses, we computed six different multiple linear regression models of which three were focused on the predictors of depression and three on the predictors of anxiety. Our findings indicated that those who experienced multiple stressful educational events were more likely to experience higher levels of depression – which again is in line with previous mental health research on depression [6,7,15]. When CSEE and covariates were adjusted for in our models, only CSEE and ethnicity were associated with higher levels of depression. Therefore, our study provides more evidence that ethnicity plays a key role in predicting mental health in educational settings [35]. Finally, when we adjusted for cognitive factors (coping and lower attentional skills), both factors were associated with higher levels of depression which provides further evidence for the association between poor coping skills and depression [23–25] as well as attention and depression [26]. Crucially, these findings are novel in the literature of doctoral students’ mental health. They provide further insight on understanding how those with poorer coping skills are more likely to experience higher levels of depression as well as those with lower attentional skills, suggesting that additional support in these skillsets may benefit doctoral students’ experience during the pandemic. Similar to the work of other studies [23,25,26], coping skills can play a key role in the experience of mental health. However, other demographic factors such as age and gender were not associated with depression contrary to previous studies that have highlighted gender contrasts in doctoral students [7,14] (see limitations for a detailed discussion of the demographic factors). Furthermore, being part of a group and being self-funded were not significant predictors of depression, which supports our theory that it is the accumulation of events rather than the experience of singular events, such as finances, that could lead to higher levels of mental health distress. Taking a closer look at the models for the stressful educational events separately (Supplementary Table 2), this is also highlighted by our data as some of the covariates (e.g., being part of a group) are only significant in either the depression or anxiety model when the events are not considered in an accumulated way. Nevertheless, as discussed it is important to examine doctoral students’ mental health through a more detailed and multidimensional model.

Conversely, we computed multiple linear regressions to explore the factors that are associated with anxiety during the same wave. CSEE was one of the key predictors in our fourth model for anxiety – suggesting that the more CSEE the doctoral students experienced the higher the levels of self-report anxiety. As expected, these findings support the current evidence available in the educational literature [6,7,15] as well as the experience of multiple stressful events and their impact on anxiety [20–23]. Although one of the covariate factors (ethnicity) in our depression models was significantly associated with the dependent variable, when we adjusted for covariates in the anxiety models none of remaining factors were significant. Such evidence highlights the complexity of the concept of mental health and the need for research to investigate mental health through multidimensional lenses. Mental health disorders are strongly associated with biological as well as environmental factors [2,36]. Here, we see that the accumulation of both environmental and biological factors can better explain mental health adversities. Finally, in the models where we adjusted for cognitive factors (coping and lower attentional skills) we see a similar pattern to the depression models where both factors are associated with higher levels of anxiety. Doctoral students with low attention scored lower on the anxiety scale. On the other hand, doctoral students with low coping skills experienced higher levels of anxiety. Both outcomes support past study findings [24,25].

Overall, our statistical models provide robust evidence on the effects of CSEE on doctoral students’ mental health during the Covid-19 pandemic. These findings not only replicate the outcomes of previous research, but they also add to the new evidence based on the statistical approach to consider the sum of CSEE. This result is relatively novel in the doctoral literature, and so is using coping skill levels as a predictor of mental health deterioration.

Despite the evidence that CSEE has a significant effect on students’ mental health during the Covid-19 pandemic, this study is not without limitations. First, the study uses cross-sectional data from a longitudinal survey with no pre-pandemic data on the mental health levels of doctoral students. Hence, our assumptions about the levels of mental health could only be based on the previous literature available [6,7,15]. Secondly, our findings must be considered strictly within the context of the Covid-19 pandemic and so this study highlights that further research is needed on the effects of CSEE on doctoral students’ wellbeing.

Furthermore, although we explored several different factors that could contribute to doctoral students struggling with depression and anxiety, our data were restricted for two reasons: a) we do not have specific measurements about the supervisory–student relationship which seems to be one of the leading factors that impact mental health [11] and b) we have not used a full standardised scale to measure lower attentional abilities. Hence, for the former, it is important to examine in depth the dynamics of the supervisor–student relationship considering its impact on mental health [11,18] and for the latter, a standardised method needs to be used in future studies on the measurement of attentional abilities. Finally, the sample in the present study is not representative of the population to account for all the challenges students face in higher education institutions as doctoral students. For example, researchers have demonstrated the stress and strain of Black doctoral students in Science, Technology, Engineering and Mathematics (STEM) [35] and this is not captured in our sample. Furthermore, our sample is heavily female skewed which could also be the reason behind these outcomes. Hence, it is important that future studies attempt to collect data from a more diverse population.

To the best of our knowledge, this is the first study that investigates the effects of multiple stressful educational experiences on doctoral students’ mental health during the Covid-19 pandemic. While there have been several studies around doctoral students’ mental health [7,11–14], most of them have focused on the exploration of factors rather than the consideration of a synergistic approach to it as other researchers studied in other areas [21–23]. The present findings indicate that those experiencing CSEE are likely to exhibit higher levels of depressive and anxiety symptoms, with a good proportion reporting clinical levels of depressive and anxiety symptoms (28.3% and 79.4%, respectively). In addition, through this work we provide further evidence on the effectiveness of coping skills as a protective factor of mental illness, potentially giving evidence for upskilling doctoral students with better coping skills. Specifically, we think that increasing doctoral students’ coping capabilities (e.g., maintaining a work–life balance; time management) will lead to less anxiety and depression as our models suggest. Finally, our findings also highlight the need for more research in the area and the factors that contribute to poor mental health to understand better how to prevent doctoral students from experiencing multiple stressful educational events.

## Figures and Tables

**Supplementary Table 1.** Number of participants per country

	n	%
Australia	9	3.4%
Bulgaria	1	0.4%
Cameroon	1	0.4%
Canada	8	3.0%
China	3	1.1%
Denmark	2	0.8%
Egypt	2	0.8%
France	4	1.5%
Germany	10	3.8%
Greece	10	3.8%
Hong Kong (S.A.R.)	2	0.8%
Ireland	3	1.1%
Italy	6	2.3%
Japan	2	0.8%
Kuwait	1	0.4%
Lebanon	9	3.4%
Norway	1	0.4%
Philippines	1	0.4%
Poland	2	0.8%
Portugal	1	0.4%
Qatar	2	0.8%
Romania	1	0.4%
Saudi Arabia	1	0.4%
Spain	3	1.1%
Turkey	1	0.4%
United Kingdom of Great Britain and Northern Ireland	154	58.3%
United States of America	23	8.7%

**Supplementary Table 2.** Coefficients for depression and anxiety models for specific events

								95% CI	
Models		Unstandardised	Standard Error	Standardised	t	*p*	VS-MPR*	Lower	Upper
Model 1 – Depression – CSEE	(Intercept)	8.48	4.20		2.02	0.05	2.60	0.16	16.80
	Research impacted by Covid-19	0.26	1.48	0.02	0.17	0.86	1.00	‒2.68	3.19
	Supervisor change	‒1.14	1.87	‒0.06	‒0.61	0.54	1.00	‒4.85	2.58
	Other problems	1.63	1.48	0.11	1.10	0.27	1.04	‒1.30	4.55
	Interruption from research	1.72	2.04	0.08	0.84	0.40	1.00	‒2.33	5.77
	Forced adaptation to research	0.54	1.28	0.05	0.42	0.67	1.00	‒2.00	3.08
Model 2 – Depression – CSEE and covariates	(Intercept)	11.54	6.92		1.67	0.10	1.61	‒2.20	25.27
	Research impacted by Covid-19	1.28	1.60	0.09	0.80	0.43	1.00	‒1.90	4.45
	Supervisor change	‒2.62	2.04	‒0.13	‒1.28	0.20	1.14	‒6.67	1.43
	Other problems	1.56	1.55	0.11	1.01	0.32	1.01	‒1.51	4.63
	Interruption from research	2.23	2.18	0.10	1.02	0.31	1.01	‒2.11	6.56
	Forced adaptation to research	1.03	1.36	0.09	0.76	0.45	1.00	‒1.68	3.74
	Age	‒0.12	0.11	‒0.12	‒1.09	0.28	1.03	‒0.35	0.10
	Gender	1.49	1.69	0.09	0.88	0.38	1.00	‒1.87	4.85
	Ethnicity	2.70	1.41	0.21	1.92	0.06	2.23	‒0.09	5.49
	**Part of a group**	**‒3.19**	**1.42**	**‒0.24**	**‒2.25**	0.03**	**3.79**	**‒6.01**	‒**0.37**
	Funded/self-funded	0.05	1.56	3.42e‒3	0.03	0.97	1.00	‒3.04	3.14
Model 3 – Depression, CSEE, covariates and cognitive factors	(Intercept)	‒3.24	7.59		‒0.43	0.67	1.00	‒18.32	11.84
	Research impacted by Covid-19	1.76	1.33	0.13	1.32	0.19	1.17	‒0.88	4.40
	Supervisor change	‒1.53	1.70	‒0.08	‒0.90	0.37	1.00	‒4.91	1.86
	Other problems	1.68	1.32	0.12	1.27	0.21	1.13	‒0.95	4.30
	Interruption from research	2.17	1.82	0.10	1.20	0.24	1.08	‒1.44	5.78
	Forced adaptation to research	1.47	1.14	0.12	1.29	0.20	1.14	‒0.79	3.73
	Age	‒0.11	0.09	‒0.11	‒1.16	0.25	1.06	‒0.30	0.08
	Gender	1.03	1.41	0.06	0.73	0.46	1.00	‒1.76	3.83
	Ethnicity	1.02	1.20	0.08	0.85	0.40	1.00	‒1.36	3.39
	Part of a group	0.23	1.31	0.02	0.17	0.86	1.00	‒2.37	2.83
	Funded/self-funded	0.23	1.30	0.02	0.18	0.86	1.00	‒2.36	2.82
	**Lower attentional abilities**	**0.71**	**0.12**	**0.56**	**6.02**	3.73e‒8**	**576292.79**	**0.47**	**0.94**
	Coping skills	‒0.14	0.08	‒0.14	‒1.68	0.10	1.64	‒0.31	0.03
Model 1 – Anxiety – CSEE	(Intercept)	5.41	3.38		1.60	0.11	1.50	‒1.29	12.11
	Research impacted by Covid-19	0.30	1.19	0.03	0.25	0.80	1.00	‒2.06	2.67
	Supervisor change	‒0.38	1.51	‒0.02	‒0.25	0.80	1.00	‒3.37	2.61
	Other problems	0.46	1.19	0.04	0.39	0.70	1.00	‒1.90	2.81
	Interruption from research	0.20	1.64	0.01	0.12	0.90	1.00	‒3.06	3.46
	Forced adaptation to research	0.71	1.03	0.07	0.69	0.49	1.00	‒1.33	2.76
Model 2 – Anxiety – CSEE and covariates	(Intercept)	8.72	5.51		1.58	0.12	1.47	‒2.22	19.66
	Research impacted by Covid-19	0.56	1.27	0.05	0.44	0.66	1.00	‒1.97	3.09
	Supervisor change	‒1.46	1.62	‒0.10	‒0.90	0.37	1.00	‒4.68	1.77
	Other problems	‒0.08	1.23	‒7.38e‒3	‒0.07	0.95	1.00	‒2.53	2.36
	Interruption from research	1.08	1.74	0.06	0.62	0.54	1.00	‒2.37	4.53
	Forced adaptation to research	0.64	1.09	0.07	0.59	0.56	1.00	‒1.51	2.80
	Age	‒0.11	0.09	‒0.13	‒1.17	0.25	1.07	‒0.29	0.07
	**Gender**	**2.71**	**1.35**	**0.21**	**2.02**	0.05**	**2.57**	**0.04**	**5.39**
	Ethnicity	0.51	1.12	0.05	0.45	0.65	1.00	‒1.72	2.73
	Part of a group	‒1.62	1.13	‒0.16	‒1.43	0.15	1.27	‒3.86	0.62
	Funded/self-funded	‒0.95	1.24	‒0.08	‒0.77	0.45	1.00	‒3.41	1.51
Model 3 – Anxiety, CSEE, covariates and cognitive factors	(Intercept)	‒2.53	6.04		‒0.42	0.68	1.00	‒14.53	9.47
	Research Impacted by Covid-19	0.96	1.06	0.09	0.90	0.37	1.00	‒1.15	3.06
	Supervisor change	‒0.61	1.36	‒0.04	‒0.45	0.65	1.00	‒3.30	2.08
	Other problems	‒5.61e‒3	1.05	‒4.99e‒4	‒5.34e‒3	1.00	1.00	‒2.09	2.08
	Interruption from research	1.05	1.45	0.06	0.73	0.47	1.00	‒1.82	3.92
	Forced adaptation to research	0.99	0.90	0.11	1.09	0.28	1.03	‒0.81	2.78
	Age	‒0.09	0.08	‒0.12	‒1.26	0.21	1.12	‒0.24	0.05
	Gender	2.34	1.12	0.19	2.09	0.04	2.87	0.11	4.56
	Ethnicity	‒0.82	0.95	‒0.08	‒0.86	0.39	1.00	‒2.71	1.07
	Part of a group	1.11	1.04	0.11	1.07	0.29	1.03	‒0.96	3.18
	Funded/self-funded	‒0.83	1.04	‒0.07	‒0.80	0.42	1.00	‒2.89	1.23
	**Lower attentional abilities**	**0.56**	**0.09**	**0.57**	**6.01**	3.89e‒8**	**554670.12**	**0.38**	**0.75**
	Coping skills	‒0.12	0.07	‒0.16	‒1.86	0.07	2.04	‒0.26	8.77e‒3

*Vovk–Sellke maximum *P*-ratio: Based on the *P*-value, the maximum possible odds in favour of H_1_ over H_0_ equals 1/(-e *P* log(*P*)) for *P* ≤ o.37.

**Statistically significant coefficients.

**Supplementary Table 3.** Multiple linear regression outcomes

Models	Multiple linear regression outputs
Model 1 – Depression	F(5,111) = 0.54, *P* < 0.75, R2 =. 03, R2 adjusted = ‒0.02
Model 2 – Depression and covariates	F(10,103) = 1.42, *P* < 0.18, R2 =0. 13, R2 adjusted =. 04
Model 3 – Depression, covariates and cognitive factors	F(12,102) = 5.32, *P* = 1.01e.6, R2 = 0.42, R2 adjusted = 0.34
Model 4 – Anxiety	F(5,114) = 4.79, *P* <. 98, R2 = 0.01, R2 adjusted = ‒0.04
Model 5 – Anxiety and covariates	F(10,103) = 0.84, *P* <0. 59, R2 =0. 08, R2 adjusted = ‒0.02
Model 6 – Anxiety, covariates and cognitive factors	F(12,102) = 4.72, *P* = 6.26e‒6, R2 =. 39, R2 adjusted = 0.30

## Data Availability

The datasets generated during and/or analysed during the current study are available in the repository: http://www.doi.org/10.5522/04/16583861.
